# Optimal Design of River Monitoring Network in Taizihe River by Matter Element Analysis

**DOI:** 10.1371/journal.pone.0127535

**Published:** 2015-05-29

**Authors:** Hui Wang, Zhe Liu, Lina Sun, Qing Luo

**Affiliations:** 1 Key Laboratory of Regional Environment and Eco-Remediation, Ministry of Education, Shenyang University, Shenyang, Liaoning Province, 110044, China; 2 Key Laboratory of Pollution Ecology and Environment Engineering, Institute of Applied Ecology, Chinese Academy of Sciences, Shenyang, Liaoning Province, 110016, China; University of Catania, ITALY

## Abstract

The objective of this study is to optimize the river monitoring network in Taizihe River, Northeast China. The situation of the network and water characteristics were studied in this work. During this study, water samples were collected once a month during January 2009 - December 2010 from seventeen sites. Futhermore, the 16 monitoring indexes were analyzed in the field and laboratory. The pH value of surface water sample was found to be in the range of 6.83 to 9.31, and the average concentrations of NH_4_^+^-N, chemical oxygen demand (COD), volatile phenol and total phosphorus (TP) were found decreasing significantly. The water quality of the river has been improved from 2009 to 2010. Through the calculation of the data availability and the correlation between adjacent sections, it was found that the present monitoring network was inefficient as well as the optimization was indispensable. In order to improve the situation, the matter element analysis and gravity distance were applied in the optimization of river monitoring network, which were proved to be a useful method to optimize river quality monitoring network. The amount of monitoring sections were cut from 17 to 13 for the monitoring network was more cost-effective after being optimized. The results of this study could be used in developing effective management strategies to improve the environmental quality of Taizihe River. Also, the results show that the proposed model can be effectively used for the optimal design of monitoring networks in river systems.

## Introduction

As an essential resource, water plays an increasingly important role in the development of economy and society. In order to protect water, the water quality monitoring, which describes the general state of water quality, started in the 1960s [1]. At that time, the monitoring network usually based on subjective criteria, and little attention was paid on the re-assessment and optimization of established monitoring network [2–3]. Since the 1970s, the studies on the water quality monitoring network had been paid more attention to [4]. The basic design criteria began to be studied in the 1980s [5], and Groot and Schilperoort (1984) discussed about the optimization [6]. Subsequently, a large number of theories and methods have been applied to the research and optimization of water quality monitoring, such as integer programming [7], multi-objective programming [8–9], entropy and generalized least-square methods [10], a methodology using geographical information system [11], fuzzy logic approach [12], *etc*. A series of efforts had been carried out since 1985 in China too, such as mean divagation [13], and similarity method [14]. But there is no universally accepted quantitative optimized method, especially the method on determination of the optimum section.

Taizihe River, located in the east of Northeast of China, is an important anabranch of Liaohe River. It provides water to Benxi, Liaoyang and Anshan for drinking, industrial and crop production. In order to strengthen the environmental protection, the monitoring network in Taizhihe River was set up in 1980s, but the re-assessment and optimization of water quality monitoring network of Taizihe River has not yet been realized. So a series of research to optimize the water quality monitoring network in Taizihe River is practically necessary to be conducted.

The aim of the present study is to develop a method to optimize the water quality monitoring network in order to support the environment monitoring of water quality management and to save the cost of monitoring on the basis of satisfying the objectives of tracking water quality distribution and variations. The matter element analysis and gravity distance have been used as an optimized model and the correlation between tributary monitoring section and adjacent mainstream monitoring section has been discussed. The model has been applied to Taizihe River in Northeast China, in order to improve the water quality monitoring sections for Taizihe River.

## Materials and Methods

### Description of the study area

Taizihe River, an important anabranch of Liaohe River, is located in the east of Northeast of China between approximately latitudes 40°29' N–41°39' N and longitudes 122°26' E–124°53' E [15]. The River drains an area of about 13,883 km^2^; its main stream is 413 km long [16]. It runs through the cities of Benxi, Liaoyang and Anshan. It joins together with Hunhe River into Daliaohe River in Sanchakou and enters to the Bohai Sea at last in Yingkou of Liaoning province. The mean annual runoff of Taizihe River is 3.68 billion m^3^, and per capita water resources (750 m^3^) are lower than per capita of the world [17].

Taizihe River flows through the central area of Liaoning Province which is industrial and agricultural production base. Benxi and Anshan are the famous iron and steel industry base in China. There are three large-scale iron and steel enterprise in Benxi City, Ansteel Group Corporation, Benxi Iron & Steel Group Co. Ltd. and Beitai Iron & Steel Group Co. per capita Ltd., which is one of the biggest iron and steel industries in China. It provides water to Benxi, Liaoyang and Anshan for drinking, industrial and crop production and the situation of water environment directly influences the local economy, so the water quality monitoring network of Taizihe River plays an essential rule in local economy.

**Fig 1:**
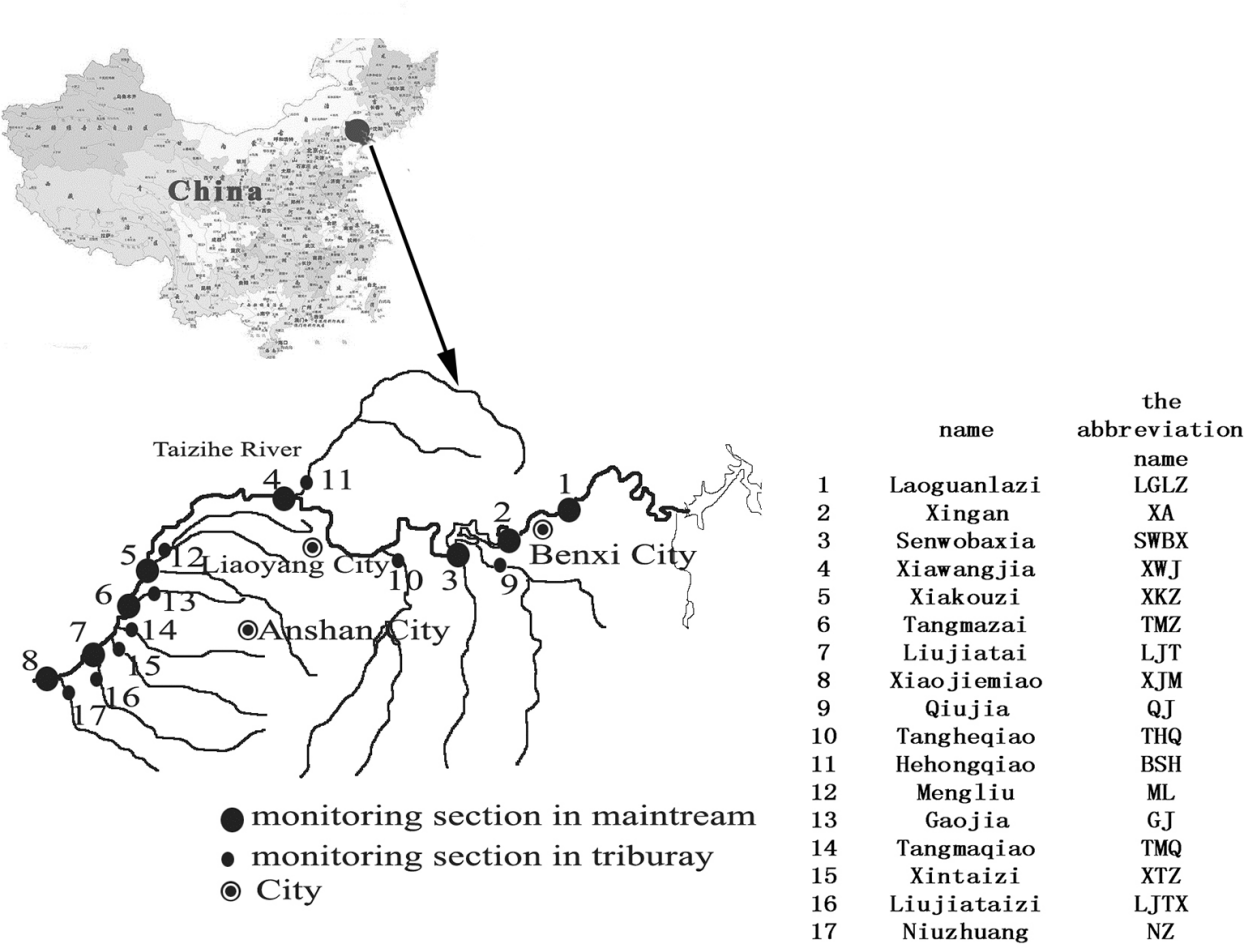
Locations of the monitoring stations in Taizihe River.

The surface water quality monitoring network in Taizihe River is composed of 17 stations in total: 8 of them located in the mainstream and 9 in the tributary (Fig 1). The coordinates of sample sites ranged from 122°30' to 123°54' E and from 40°57' to 41°23' N. There were no specific permits required for the described field studies and no specific permissions required for these activities. And the field studies did not involve endangered or protected species.

### Analytical methods

Water samples were collected once a month during January 2009 to December 2010 from seventeen sites, and the measurement of the monitoring indexes was performed in the field and laboratory. The monitoring indexes are shown in Table 1. Three samples at each station were collected from a depth of 1 foot below the surface of running water, using a 500 ml polythene or glass bottle. The samples were stored in an ice box and later refrigerated to 4°C before being analyzed in the laboratory [18–20]

**Table 1:** Water characteristics of Taizihe River for data collected at seventeen sites between 2009 and 2010.

Indexes	2009		2010		
	Mean ±S.D.	Range	Mean ±S.D.	Range	Standard
NH_4_^+^-N (mg/L)	5.428±6.955	0.025–73.330	4.094±4.256	0.025–16.000	≤1.0
COD(mg/L)	28.703±23.376	5–172	21.535±17.771	5–96.1	≤20.0
volatile phenol(mg/L)	0.013±0.061	0.001–0.853	0.008±0.022	0.001–0.240	≤0.005
pH	7.93±0.42	6.83–9.31	7.93±0.39	6.50–9.00	6.0–9.0
DO(mg/L)	6.85±2.89	0.10–13.97	7.10±2.95	0.34–14.6	≥5
TP(mg/L)	0.392±0.360	0.005–1.580	0.292±0.298	0.005–1.330	≤0.2
Cu(mg/L)	0.0073±0.0067	0.005–0.045	0.0052±0.0015	0.005–0.019	≤1.0
Cyanide (mg/L)	0.0096±0.0241	0.0019–0.142	0.0117±0.0272	0.002–0.17	≤0.2
Zn(mg/L)	0.032±0.026	0.025–0.216	0.034±0.035	0.025–0.340	≤1.0
fluoride(mg/L)	0.659±0.445	0.11–2.32	0.761±0.661	0.02–3.67	≤1.0
Se(μg/L)	2.00±0.03	1.87–2.31	2.00±0.03	1.70–2.20	≤10
As(μg/L)	4.00±0.02	3.90–4.20	3.99±0.02	3.76–4.10	≤50
Cd(μg/L)	0.50±0.05	0.44–1.00	0.53±0.34	0.50–4.90	≤5
Cr(μg/L)	2.00±0.05	1.70–2.40	2.00±0.04	1.70–2.30	≤50
Hg(μg/L)	0.044±0.059	0.02–0.44	0.031±0.041	0.02–0.49	≤0.1
Pb(μg/L)	5.00±0.04	4.80–5.50	5.00±0.02	4.90–5.23	≤5

The portable equipments (HACH HQ40D) were used to measure dissolved oxygen (DO) and pH immediately on spot [19]. The ammonia (NH_4_^+^-N), TP and COD were assessed by Kenker325, Kenker323 and Kenker83212, respectively. Other water monitoring indexes were acquired in the laboratory using the recommended test methods [21]. Heavy metal contents (Cu, Zn, Cr, Hg, Pb and Cd) were determined with an atomic-absorption spectrophotometer Spectr AA 220 [20], and the standard stock solution of soluble salts was used for quantifying the samples [22]. A hydride generation AAS was used to determine arsenic (As) in water [23–24].

Differences of the obtained data between sampling sites were tested using analysis of variance (ANOVA), processed by the SPSS statistics software 15.0 for Windows. The results of all indices represent the average of the three samples for each site.

### Present situation analysis of river monitoring network

There are some basic features in reasonable designed monitoring network, such as the higher data availability and the lower correlation between adjacent sections [25]. So the data availability and the correlation between adjacent sections were used to analyze the present situation of river monitoring network.

#### Data availability of monitoring section

The data availability of monitoring section (a), as a measurable indicator of the situation of river monitoring network, was used to describe the ratio of monitoring data acquired in the monitoring practice and its standard was 90% [26–27]. It can be calculated as:

$$\alpha =\frac{\sum_{1}^{s}p_{i}}{RT}$$ (1)

where: P is the monitoring frequency in the monitoring practice; R is the monitoring frequency in the expected monitoring; S is the amount of monitoring indexes in the monitoring practice; and T is the amount of monitoring indexes in the expected monitoring.

#### Correlation between adjacent sections

The duplicate setting of monitoring section may overemphasize the importance of some monitoring indexes and affect the accuracy of monitoring network. The correlation between adjacent sections (r) was an important index to reflect the duplicate setting degree of monitoring sections [25–26]. It can be calculated as:

$$r=\frac{l_{xy}}{\sqrt{l_{xx}l_{yy}}}$$ (2)

lxx=∑1nxi2−(∑1nxi)2n (3)

lyy=∑1nyi2−(∑1nyi)2n (4)

lxy=∑1nxiyi−∑1nxi•∑1nyin (5)

where: r is the correlation between adjacent sections; n is the number of years when the monitoring data acquired; x_i_ and y_i_ are the mean value of one major pollutant between two adjacent sections; *l*_*xx*_ and *l*_*yy*_ are the sum of squares of mean deviation of x and y; and *l*_*xy*_ is the deviation from mean of x and y. The value of r_0.05_ and r _0.01_ were read form the correlation coefficient table of critical values. If r ≤ r_0.05_, it meaned that no significant correlation was found between adjacent sections; if r_0.05_ ≤ r ≤ r_0.01_, the correlation between adjacent sections was significant; otherwise, the correlation was very significant.

### Matter element analysis

Matter element analysis, established by Cai in 1976, was a subject between mathematics and experimental science [28]. It has been applied in optimized sites of atmospheric monitoring by Zhu and Yu in 1998 [29] as well as in optimized points selection of water quality environmental monitoring by Gao in 1997 [30]. But the application of matter element analysis in optimized points selection has not yet been well developed.

#### The establishment of the matter element matrix

According to the detection value of all water monitoring indexes, the most ideal point (a), the least ideal point (b) and the mathematical expectation point (c) were obtained using the following method:

$$\text{the most ideal point}\;\left(\text{a}\right):\;\left\{\begin{array}{c} \text{min}\\ i \end{array}x_{ij},j\in J;\begin{array}{c} \text{max}\\ i \end{array}x_{ij},j\in J'\right\}$$

$$\text{the least ideal point}\;\left(\text{b}\right):\;\left\{\begin{array}{c} \text{max}\\ i \end{array}x_{ij},j\in J;\begin{array}{c} \text{min}\\ i \end{array}x_{ij},j\in J'\right\}$$

$$\text{the mathematical expectation point}\;\left(\text{c}\right):\;\left\{{\bar{x}}_{j}=\sum_{i=1}^{n}x_{ij}/n\right\}$$

where: *x*_*ij*_ is the detection value of j^th^ water monitoring indexes in the i^th^ monitoring section; n is the number of monitoring sections; *J* is a set of positive index and *J*’ was a set of reverse index.

Matter element matrix of standard (*R*_*ac*_ and *R*_*cb*_) and partial unit (*R*_*ab*_) can be established on condition that the optimum and the worst points (a and b) are chosen [30]. The matter element matrix *R*_*ac*_ comprises of variable ranges of water monitoring indexes of point a and point b. The matter element matrix *R*_*cb*_ comprises of variable range of water monitoring indexes of point c and point b. And the matter element matrix *R*_*ab*_ comprises of variable range of water monitoring indexes of point a and point b.

$$R_{ac}=\left[M_{ac},\left(\begin{array}{ccc} Q_{1}<a_{1} & , & c_{1}>\\ \vdots & \ddots & \vdots \\ Q_{m}<a_{m} & , & c_{m}> \end{array}\right)\right]$$ (6)

$$R_{cb}=\left[M_{cb},\left(\begin{array}{ccc} Q_{1}<c_{1} & , & b_{1}>\\ \vdots & \ddots & \vdots \\ Q_{m}<c_{m} & , & b_{m}> \end{array}\right)\right]$$ (7)

$$R_{ab}=\left[M_{ab},\left(\begin{array}{ccc} Q_{1}<a_{1} & , & b_{1}>\\ \vdots & \ddots & \vdots \\ Q_{m}<a_{m} & , & b_{m}> \end{array}\right)\right]$$ (8)

Taking the monitoring section as a preparative optimization unit, the detection value of monitoring section formed the matter element matrix of preparative optimization unit (*R*_*i*_).

$$R_{i}=\left[M_{i},\left(\begin{array}{ccc} Q_{1} & & X_{i}{}_{1}\\ \vdots & & \vdots \\ Q_{m} & & X_{i}{}_{m} \end{array}\right)\right]$$ (9)

#### Determination of classification during sections

Based on matter element matrix of standard (*R*_*ac*_ and *R*_*cb*_) and partial unit (*R*_*ab*_), the related function value (*Ka(x*_*i*_ *)* and *K*_*b*_ *(x*_*i*_ *)*) regarding standard substance element with the monitoring point were calculated using the following methods:

$$K_{a}(x_{ij})=\frac{x_{ij}-c_{j}}{c_{j}-a_{j}}$$ (10)

$$K_{b}(x_{ij})=\frac{x_{ij}-c_{j}}{c_{j}-b_{j}}$$ (11)

$$K_{a}(x_{i})=\sum_{j=1}^{m}W_{j}K_{a}(x_{ij})$$ (12)

$$K_{b}(x_{i})=\sum_{j=1}^{m}W_{j}K_{b}(x_{ij})$$ (13)

where: *Ka(x*_*ij*_ *)* and *Kb(x*_*ij*_ *)* are the related function value of j^th^ water monitoring indexes in the i^th^ monitoring section; *W*_*j*_ is the weighting coefficient of j^th^ water monitoring indexes.

Assuming (*Ka(x*_*i*_ *)* and *K*_*b*_ *(x*_*i*_ *)*) as coordinate, the scatter diagram of monitoring sections are plotted. According to the distribution of monitoring sections in the scatter diagram, the classification of sections is determined.

#### Determination of the optimum section

After the classification of sections was determined, then center of gravity (G) of every classification was calculated.

$${\text{G}}_{\text{i}}=\left\{{\text{G}}_{\text{i}1},{\text{G}}_{\text{i}2},\;\ldots ,\;{\text{G}}_{\text{ij}}\right\}$$

where: G_i_ is the center of gravity of the classification i; G_ij_ is the detection value of j^th^ water monitoring indexes of G_i_.

$$G_{ij}=\frac{\sum_{k=1}^{M}y_{ijk}}{M}$$ (14)

where: M is the amount of sections in the classification I; y_ijk_ is the detection value of j^th^ water monitoring indexes of monitoring sections K (_yik_) in the classification i.

Euclidean distance (R_ik_) between G_i_ and monitoring sections K was calculated with the following formula:

$$R_{ik}={\left[\sum_{j=1}^{N}{\left(y_{ijk}-G_{ik}\right)}^{2}\right]}^{1/2}$$ (15)

where: R_ik_ is the Euclidean distance (R_ik_) between Gi and K; N is the amount of monitoring indexes in every sections.

The smaller R_ik_ was, the more the representative of sections K to the classification i. So if the adjacent monitoring sections in mainstream were in the same classification, the section with smaller R_ik_ should be retained and the one with bigger R_ik_ should be removed. If the monitoring section in one tributary and the following adjacent monitoring section in mainstream were in the same classification, the monitoring section in tributary should be removed.

### Statistical analysis

The experimental data were analyzed by SPSS 12.0 for Windows. Excel 2003 and Origin Pro 7.5 were used to plot the experimental data. The results of all indices represent the average of the three samples for each site.

## Results and Discussions

### Water characteristics of Taizihe River

The mean, standard deviation and the range of variation for each monitoring indexe of Taizihe River were summarized in Table 1. In the majority of the sampled points, the physical parameters showed a low variability during the studied period. The pH value varied from 6.83 to 9.31, with a mean value of 7.93±0.42 in 2009, and it varied from 6.50 to 9.00, with a mean of 7.93±0.39 in 2010; the DO from 0.10 to 13.97,with an average of 6.85±2.89 in 2009, and from 0.34 to 14.6, with an average of 7.10±2.95 in 2010. Opposite to the physical parameters, the chemical parameters showed a high variability during the studied periods, except heavy metal.

According to the Ⅲ level of Environmental quality standards for surface water (GB3838-2002)(EQSSQ) of China, the concentrations of NH_4_^+^-N, COD, volatile phenol and TP in most of the sampled points were found to be above the standard values, while other parameters could usually meet the standard. NH_4_^+^-N, COD, volatile phenol and TP were the main pollutants in the surface water of Taizihe River. Similar results were also observed by Yang in 2002 [31] and Li *et al*. in 2011 [32] in Taizihe River. Based on the comparative analysis about the variation of water quality of the main sections of the Taizihe River during high-water periods and drought periods from 1991 to 2000, Yang found out that COD, NH_4_^+^-N, volatile phenol were the main pollutants in 2002 [31].

From 2009 to 2010, the average concentrations of NH_4_^+^-N, COD, volatile phenol and TP had decreased significantly. Meanwhile, the mean concentrations of cyanide and fluoride have slightly increased from 0.0096 mg/L and 0.659 mg/L in 2009 to 0.0117 mg/L and 0.761 mg/L in 2010, respectively. For the heavy metals, there were no significant changes in the concentrations of Zn, Cd, Cr and Pb; whereas in case of Cu and Hg, the average concentrations have decreased. In a word, the surface water quality showed an upward turn in 2010 compared with 2009.

### Present situation of river monitoring network in Taizihe River

The data availability of monitoring section in Taizihe River was shown in Table 2. In 2009, only the data availabilities of four mainstream monitoring sections reached 90%. The data availability was better in 2010 than in 2009, and there were ten monitoring sections where the data availability reached 90%. The ratio of monitoring data acquired was lower than the expectation in some degree, so the monitoring sections should be optimized. The precipitation changed significantly among different periods and different areas in Liaoning Province. The precipitation ratio was 64.8% in summer, while it was only 2.75% in winter [33]. At the same time, the precipitation gradually diminished from southeast to northwest direction of Liaoning Province [34]. It was a reason of the lower data availabilities in Taizihe River.

**Table 2:** Data availability of monitoring sections in Taizi River.

Monitoring sections	Year	The actual monitoring frequency	The frequency of monitoring plan	The total number of years of practical monitoring project	The total number of years of planning monitoring project	Data availability
LGLZ	2009	12	12	276	23	1.00
	2010	12	12	276	23	1.00
XA	2009	12	12	275	23	1.00
	2010	12	12	272	23	0.99
SWBX	2009	12	12	234	23	0.85
	2010	12	12	264	23	0.96
XWJ	2009	12	12	252	23	0.91
	2010	12	12	264	23	0.96
XKZ	2009	12	12	234	23	0.85
	2010	12	12	264	23	0.96
TMZ	2009	12	12	192	23	0.70
	2010	12	12	264	23	0.96
LJT	2009	12	12	192	23	0.70
	2010	12	12	256	23	0.93
XJM	2009	12	12	253	23	0.92
	2010	10	12	220	23	0.80
QJ	2009	12	12	133	23	0.48
	2010	12	12	195	23	0.71
THQ	2009	12	12	234	23	0.85
	2010	12	12	264	23	0.96
BSH	2009	12	12	234	23	0.85
	2010	12	12	264	23	0.96
ML	2009	12	12	234	23	0.85
	2010	12	12	264	23	0.96
GJ	2009	12	12	132	23	0.48
	2010	11	12	184	23	0.67
TMQ	2009	12	12	132	23	0.48
	2010	12	12	194	23	0.70
XTZ	2009	12	12	132	23	0.48
	2010	12	12	194	23	0.70
LJTZ	2009	12	12	132	23	0.48
	2010	10	12	165	23	0.60
NZ	2009	12	12	132	23	0.48
	2010	12	12	194	23	0.70

The correlation of NH_4_^+^-N between neighboring monitoring sections in main stream of Taizihe River was shown in Table 3. The correlations of NH_4_^+^-N were significant between four groups of adjacent sections, such as SWBX—XWJ, XWJ—XKZ, TMZ—LJT and LJT—XJM. And there were no significant correlations between three groups of adjacent section, such as LGLZ—XA, XA—SWBX and XKZ—TMZ. So There was duplicate setting between monitoring sections in main stream of Taizihe River, and the optimal design of monitoring network is thus necessary in Taizihe River.

**Table 3:** The correlation of NH_4_^+^-N between neighboring monitoring sections in main stream of Taizihe River.

Adjacent monitoring sections	The correlation of NH4+-N
LGLZ—XA	No significant
XA—SWBX	No significant
SWBX—XWJ	significant
XWJ—XKZ	significant
XKZ—TMZ	No significant
TMZ—LJT	significant
LJT—XJM	significant

### Optimization of water quality monitoring networks by matter element analysis

**Fig 2:**
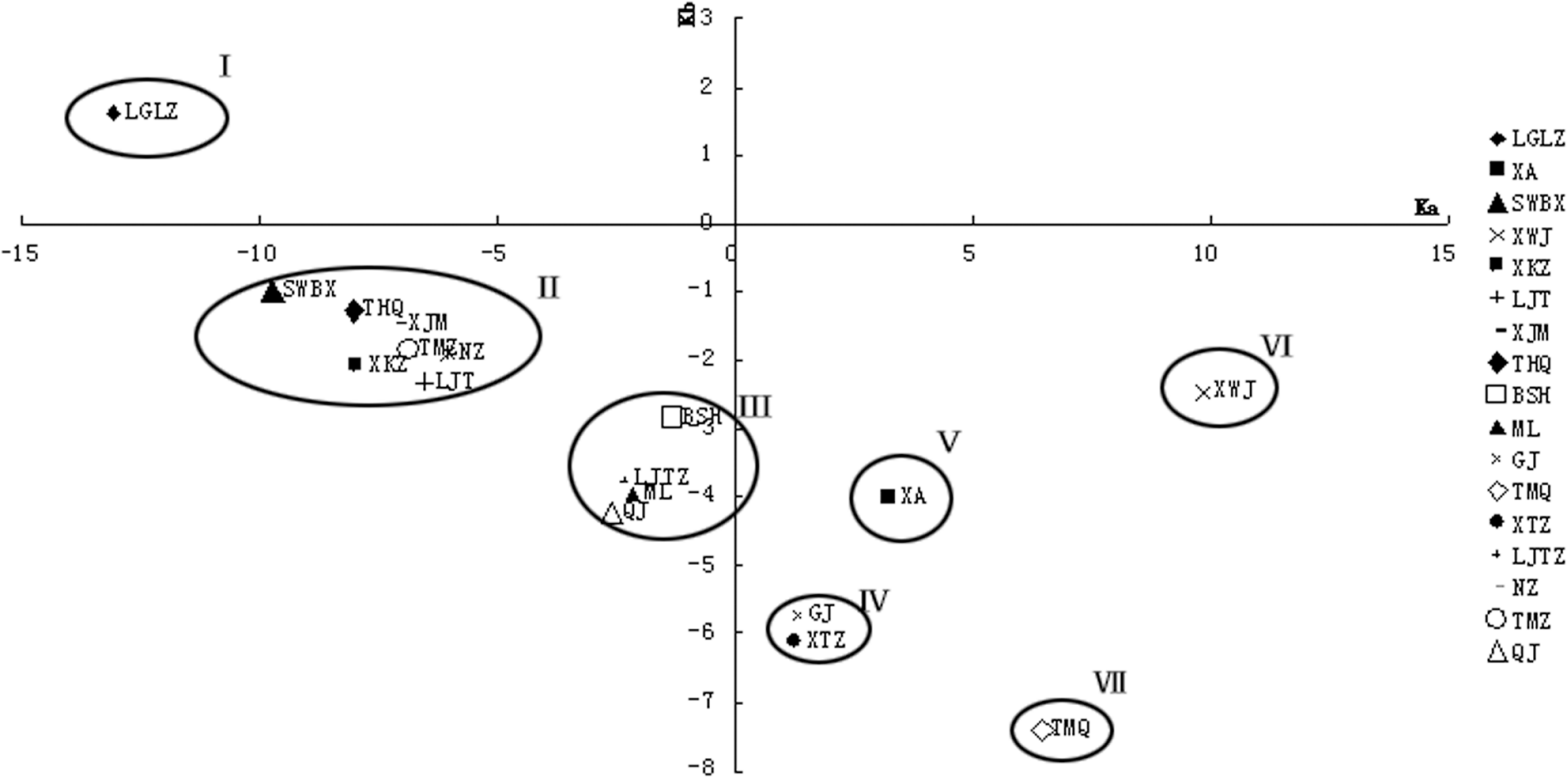
The result of matter element analysis in main stream of Taizihe River.

Based on the water quality monitoring data from January 2009 to June 2010, the present water quality monitoring networks were optimized by matter element analysis in Taizihe River. According to matter element model, the most ideal point (a), the least ideal point (b) and the mathematical expectation point (c) in Taizihe River were obtained, and listed in Table 4. According to the formula 6–13, *Ka(x*_*i*_ *)* and *K*_*b*_ *(x*_*i*_ *)* of the monitoring sections in Taizihe River were calculated. Then taking *Ka(x*_*i*_ *)* as the X coordinate and *K*_*b*_ *(x*_*i*_ *)* as the Y coordinate, the scatter diagram of monitoring sections were plotted (Fig 2). The classification and the euclidean distance (R_ik_) between the section and the center of gravity of its classification were shown in Table 5. In classification Ⅰ, Ⅴ, Ⅵ and Ⅶ, there was only one monitoring section in every classification. So LGLZ, XA, XWJ and TMQ should be retained. LGLZ, XA, XWJ were monitoring sections of mainstream and TMQ was a monitoring section of tributary. There were seven monitoring sections in classification Ⅱ. Five monitoring sections, SWBX, XJM, TMZ, XKZ and LTJ, were in mainstream and two monitoring sections, THQ and NZ, were in tributary. The R_ik_ of NZ was the highest (17.199) and the R_ik_ of XJM was the lowest (2.229) among the monitoring sections from upstream to downstream in classification Ⅱ. At the same time, XKZ, TMZ, LTJ and XJM were the adjacent monitoring sections in mainstream. So XJM was retained, and XKZ, TMZ and LTJ would be removed. XJM was the adjacent mainstream monitoring section of NZ (the tributary monitoring section), and the R_ik_ of NZ was higher than XJM. So NZ was removed. BSH, LJTX, ML and QJ were in classification Ⅲ. The R_ik_ of LJTX was the highest and the R_ik_ of ML was the lowest. BSH, LJTX, ML and QJ were located in different tributary, so they would be retained. There were two sections, GJ and XTZ, in classification Ⅳ. The R_ik_ of GJ and XTZ were the same, and they located in different tributaries. So GJ and XTZ were retained.

**Table 4:** The numerical value of the most ideal point (a), the least ideal point (b) and the mathematical expectation point (c).

Indexes	the optimum point (a)	the worst point (b)	the mathematical expectation point (c)
NH_4_^+^-N (mg/L)	0.05	11.11	4.77
COD(mg/L)	5.00	51.89	25.22
volatile phenol(mg/L)	0.0009	0.0588	0.0105
pH	7.62	8.33	7.93
DO(mg/L)	3.40	11.19	6.97
TP(mg/L)	0.02	0.83	0.33
Cu(mg/L)	0.004	0.008	0.006
Cyanide (mg/L)	0.002	0.061	0.012
Zn(mg/L)	0.025	0.071	0.032
fluoride(mg/L)	0.146	2.438	0.850
Se(μg/L)	0.002	0.002	0.002
As(μg/L)	0.004	0.004	0.004
Cd(μg/L)	0.00050	0.00052	0.00050
Cr(μg/L)	0.002	0.002	0.002
Hg(μg/L)	0.00002	0.00009	0.00004
Pb(μg/L)	0.005	0.005	0.005

**Table 5:** The Euclidean distance (R_ik_) between the section and the center of gravity of its classification.

Classification	Monitoring section	the Euclidean distance (Rik)
I	LGLZ	0
II	SWBX	12.617
	THQ	11.093
	XJM	3.229
	TMZ	2.477
	NZ	17.199
	XKZ	3.258
	LTJ	4.469
III	BSH	5.009
	LJTX	18.240
	ML	4.866
	QJ	10.400
IV	GJ	3.702
	XTZ	3.702
V	XA	0
VI	XWJ	0
VII	TMQ	0

The number of monitoring sections drops from 17 to 13 after using matter element analysis and gravity distance. There were five monitoring sections in mainstream, *i*.*e*. LGLZ, XA, SWBX, XWJ and XJM, and there were eight monitoring sections in tributary, *i*.*e*. THQ, BSH, LJTX, ML, GJ, QJ, XTZ and TMQ. 23.52% of the cost of monitoring can be saved and the monitoring network was more effective after optimization.

### The verification on the result of water quality monitoring optimization

Based on the water quality monitoring data from July 2010 to December 2010, the difference between un-optimized monitoring networks and optimized monitoring networks were compared by F-test (variance test) and T-test (mean test). Significance level was 0.05, and the main pollutants (COD, NH_4_^+^-N and volatile phenol) were chosen as sample variable. The results of the test were shown in Table 6. The results of F-test on COD, NH_4_^+^-N and volatile phenol were homoscedastic and the results of T-test on the main pollutants have no significant differences. There were no significant differences between non-optimized monitoring networks and optimized monitoring networks. So the optimized monitoring networks by matter element analysis could correctly represent the original monitoring network. At the same time, it was highly-efficient and more economic.

**Table 6:** The test on the result of water quality monitoring optimization in Taizi River.

Sample variable	F-test			T-test			
	F	Sig.	result	t	Lowwer limit	upper limit	result
NH4_+_-N	0.08	0.783	homogeneity of variance	0.325	2.06964	2.77702	NSD
COD	0.03	0.866	homogeneity of variance	0.44	7.53787	11.25243	NSD
volatile phenol	0.019	0.893	homogeneity of variance	0.479	.1.12345	1.73868	NSD

## Conclusions

This paper has provided data on river monitoring network of the Taizihe River, which located in the heavy industrial area in the northeast of China. It was found that the pH value varied from 6.83 to 9.31 and the average concentrations of NH_4_^+^-N, COD, volatile phenol and TP decreased significantly. NH_4_^+^-N, COD, volatile phenol and TP were the main pollutants in the surface water of Taizihe River. Through the calculation of the data availability and the correlation between adjacent sections, it was found that there was duplicate setting between monitoring sections of Taizihe River; and the optimal design of monitoring network was necessary. The application of the matter element analysis and gravity distance in Taizihe River confirmed that the matter element model and gravity distance is an efficient method to optimize river quality monitoring network. The number of monitoring sections cut from 17 to 13after the optimization by using matter element analysis and gravity distance. 23.52% of the cost of monitoring was saved and the monitoring network was more effective after being optimized. This information of this study could be used to develop effective management strategies to control and manage the river quality of Taizihe River. And it provided an effective method to optimize river quality monitoring network.

## References

[1] Strobl RO, Robillard PD (2008) Network design for water quality monitoring of surface freshwaters: A review. Journal of Environmental Management 87: 639–648. 17459570 10.1016/j.jenvman.2007.03.001

[2] Ward RC (1996) Water quality monitoring: Where's the beef?. water resources bulletin 32(4): 673–680.

[3] Harmancioglu NB, Alpaslan MN (1992) Water quality monitoring network design. J Am Water Resour Assoc 28: 179–192.

[4] Beckers CV, Chamberlain SG (1974) Design of Cost-effective Water Quality Surveillance Systems. US Environmental Protection Agency, Washington DC.

[5] Dixon W, Chiswell B (1996) Review of aquatic monitoring program design. Water Res 30(9): 1935–1948.

[6] Groot S, Schilperoort T (1984) Optimization of water-quality monitoring networks. Water Sci Technol 16(5): 275–287.

[7] Hudak PF, Loaiciga HA, Marino MA (1995) Regional-scale ground-water quality monitoring via integer programming. J Hydrol 164(1):153–170.

[8] Cieniawski SE, Eheart JW, Ranjithan S (1995) Using genetic algorithms to solve a multiobjective groundwater monitoring problem. Water Resour Res 31(2):399–409.

[9] Harmancioglu NB, Alpaslan N (1992) Water-quality monitoring network design: a problem of multiobjective decision-making. Journal of the American Water Resources Association 28(1):179–192.

[10] Markus M, Knapp HV, Tasker GD (2003) Entropy and generalized least square methods in assessment of the regional value of streamgages. Journal of Hydrology 283:107–121.

[11] Strobl RO, Robillard PD, Shannon RD, Day RL, McDonnell AJ (2006) A water quality monitoring network design methodology for the selection of critical sampling points: Part I. Environmental Monitoring and Assessment 112:137–158. 16404538 10.1007/s10661-006-0774-5

[12] Strobl RO, Robillard PD, Day RL, Shannon RD, McDonnell AJ (2006) A water quality monitoring network design methodology for the selection of critical sampling points: Part II. Environmental Monitoring and Assessment 122:319–334. 16502278 10.1007/s10661-006-0358-4

[13] Jiang X (2006) Application of mean divagation in optimizing of river quality monitoring section. Heilongjiang Environmental Journal 30(2):44–45.

[14] Guo XQ (2005) Optimized Selection in Water Quality Monitoring of River Inside the City by Similarity Method. Bulletin of Science and Technology 21(3): 360–363.

[15] Guo SP, Chen GD (2008) Application of fuzzy comprehensive appraisal in evaluation of water quality for Liaoyang block of Taizihe river. Water Resources & Hydropower of Northeast China 26(2): 50–52.

[16] Yang YJ (2002) Causes of water pollution of Taizihe River in Liaoning Province and countermeasures. Water Resources Protection 2: 14–16.

[17] Li WS, Xu SG (2007) Evaluation on sustainable utilization of water resources and countermeasures in Taizi River Basin. South-to-north Water Transfers and Water Science & Technology 5(3): 51–53.

[18] Kar D, Sur P, Mandanl SK, Saha T, Kole RK (2008) Assessment of heavy metal pollution in surface water. Int J Environ Sci Tech 5: 119–124.

[19] Ayenimo JG, Adeeyinwo CE, Amoo LA (2005) Heavy metal pollutants In Warri River, Nigeria. Kragujecac J Sci 27: 43–50.

[20] Chilundo M, Kelderman P, O'keeffe JH (2008) Design of a water quality monitoring network for the Limpopo River Basin in Mozambique. Physics and Chemistry of the Earth 33: 655–665.

[21] APHA/AWWA/WPCF (2005) Standard Methods for the Examination of Water and Wastewater, 21st edition. Washington, DC, USA.

[22] Diagomanolin V, Farhang M, Ghazi-Khansari M, Jafarzadeh N (2004) Heavy metals (Ni, Cr, Cu) in the Karoon waterway river, Iran. Toxicol Lett 151, 63–68. 15177641 10.1016/j.toxlet.2004.02.018

[23] Cong NV (1999) Monitroring and assessment of potential risk for heavy metals contamination in surface and ground water at Mae Moh Mine and Power Plant Changwat Lampang. M.Sc. Thesis, Chiang Mai University, Thailand.

[24] Junshum P, Menasveta P, Traichaiyaporn S (2007) Water quality assessment in reservoirs and wastewater treatment system of the Mae Moh Power Plant, Thailand. J Agri Soc Sci 3(3): 91–94.

[25] Xiao ZX (2008) The study on the optimization design of surface water environment monitoring sites of Huaihe River in Anhui Province. M. Sc. Thesis, Hefei University of Technology, China:8–9.

[26] Dong C (2004) Optimization of river water quality monitoring network in Shandong province. M. Sc. Thesis, Shandong University, China: 19–21.

[27] Li X (1996) Study on the rationality of China surface water environment monitoring point arrangement. M. Sc. Thesis, Northeast Normal University, China:18

[28] Zhang Y, Ni XM (2005) Application of substance-element analysis to environment quality assessment and spot optimization. Environmental Science and Technology 28(z1):114–115.

[29] Zhu HJ, Yu YP (1998) Application of Matter Element Analysis to Optimized Sites of Atmospheric Monitoring. Yunnan Environmental Science 17(4): 51–53.

[30] Gao MH (1997) Study on optimized points selection of water quality environmental monitoring by matter element analysis. Advanced in Environmental Science 5(3): 77–81.

[31] Yang YJ (2002) Causes of water pollution of Taizihe River in Liaoning Province and countermeasures. Water Resources Protection 2: 15–16.

[32] Li S, Zhou XD, Li J (2011) Research on water environmental capacity of the Taizi River basin. Journal of Water Resources and Water Engineering 22(5): 111–114.

[33] Yang WY, Chi CY (2008) Analysis on characteristics of temporal and spatial changes for precipitation In Liaoning Province. Journal of Anhui Agr Sci 36(21): 9197–9199, 9209.

[34] Li GX, Liu XF, Li JL, Wang HM, Dai YR (2010) Analysis on temporal and spatial characteristics of temperature and precipitation in Liaoning Province. Journal of Anhui Agr Sci 38(32): 18337–18342.

